# Multimodal ocular imaging of known and novel corneal stromal disorders in dogs

**DOI:** 10.1186/s12917-022-03214-7

**Published:** 2022-03-26

**Authors:** Sangwan Park, Lionel Sebbag, Bret A. Moore, M. Isabel Casanova, Brian C. Leonard, Nicole L. Daley, Kirsten A. Steele, Jennifer Y. Li, Christopher J. Murphy, Sara M. Thomasy

**Affiliations:** 1 Department of Surgical and Radiological Sciences, School of Veterinary Medicine, University of California-Davis, Davis, CA 95616 USA; 2 William R. Pritchard Veterinary Teaching Hospital, School of Veterinary Medicine, University of California-Davis, Davis, CA 95616 USA; 3 Koret School of Veterinary Medicine, The Hebrew University of Jerusalem, 76100 Rehovot, Israel; 4 Department of Small Animal Clinical Sciences, College of Veterinary Medicine, University of Florida, Gainesville, FL 32608 USA; 5 Eye Care for Animals, Reno, NV 89511 USA; 6 Department of Ophthalmology & Vision Science, School of Medicine, University of California Davis, Davis, CA 95817 USA

**Keywords:** Corneal opacity, Fourier-domain optical coherence tomography, In vivo confocal microscopy, Lipid keratopathy, Mucopolysaccharidosis, Pre-Descemet corneal dystrophy

## Abstract

**Background:**

Imaging features obtained with Fourier-domain optical coherence tomography (FD-OCT) and in vivo confocal microscopy (IVCM) for corneal stromal disorders have been sparsely reported in dogs. This case report is a compilation of imaging features for three cases of different stromal disorders of the canine cornea which have not yet been reported elsewhere.

**Case presentation:**

Lipid deposition in case 1 appeared as needle-shaped hyperreflective lines along the collagen lamellae, which correlated histologically with lipid clefts. In case 2, glycosaminoglycan accumulation by mucopolysaccharidosis type 1 caused diffuse stromal hyperreflectivity and depletion of keratocytes on IVCM and was associated with secondary corneal degeneration presumed to be calcium deposition. In case 3, posterior corneal stromal opacities in the absence of ocular inflammation were identified. Hyperreflective particles were scattered in the middle and posterior corneal stroma on FD-OCT. With IVCM, hyperreflective deposits were identified within keratocytes and the number of enlarged keratocytes containing hyperreflective deposits increased towards the posterior stroma. The bilateral, non-inflammatory nature and unique appearance with IVCM is most consistent with a posterior stromal dystrophy reminiscent of pre-Descemet corneal dystrophy described in humans.

**Conclusions:**

In vivo multimodal corneal imaging facilitated instantaneous microstructural analysis and may be valuable in the differential diagnosis of corneal stromal disorders in veterinary clinical practice. The non-specific nature of imaging findings occurs in some conditions such as mucopolysaccharidosis, thus in vivo corneal imaging should be complemented with other gold standard methods of definitive diagnosis.

## Background

Corneal transparency is maintained by avascularity, deturgescence and uniform arrangement of stromal collagen fibrils [1]. Dystrophy, degeneration, inflammation, or abnormal intracellular/extracellular inclusions could alter one of these three mechanisms, leading to corneal opacification. Historically, the diagnosis of corneal diseases has been based on signalment, history and slit lamp examination, but the condition may go undetected until the lesion reaches a critical size. As most corneal stromal opacities do not impact vision until they are severe and are not painful unless ulceration occurs, histopathology of affected corneas is rarely recommended in client-owned animals. With the expansion of in vivo corneal imaging, the microstructural characteristics of various corneal stromal abnormalities could facilitate earlier in-depth diagnosis and monitoring of disease progression [2].

Herein, we report lipid keratopathy (case 1), mucopolysaccharidosis (MPS)-affected corneas with secondary calcium degeneration (case 2) and a novel posterior stromal dystrophy (case 3), all of which were characterized with in vivo multimodal imaging. The purpose of this case series is to describe differential imaging features of the aforementioned corneal conditions that have not been reported yet in veterinary literature, and to evaluate clinical utility of corneal imaging in different disease conditions.

## Case presentation

Three dogs presented to the Comparative Ophthalmology Service of the William R. Pritchard Veterinary Medical Teaching Hospital at University of California, Davis for evaluation of corneal opacities were included in this study. All dogs had complete ophthalmic examinations, including measurements of tear production (Schirmer’s tear test (STT-1); Schering-Plough, Merck), intraocular pressure (IOP; Tonopen Avia, Reichert Technologies; Tonovet, Icare, Finland), slit lamp biomicroscopy (SL-15, Kowa, Tokyo, Japan; SL-D701, Topcon, Tokyo, Japan), indirect ophthalmoscopy (Vantage Plus, Keeler, Broomall, PA, USA; Volk 28D lens, Mentor, OH, USA) and ocular surface fluorescein staining (HUB Pharmaceuticals, CA, USA). Following ocular examination, Fourier-domain optical coherence tomography (FD-OCT; Optovue, Fremont, CA, USA) and in vivo confocal microscopy (IVCM; ConfoScan 4, Nidek, Japan; Heidelberg Retinal Tomography 3 in conjunction with the Rostock Cornea Module, Heidelberg Engineering, Dossenheim, Germany) were performed with gentle manual restraint following topical application of 0.5% proparacaine (Akorn Pharmaceuticals, Lake Forest, IL, USA). Sedation for ocular imaging was not required for all dogs. Corneal fluorescein stain was repeated to ensure that corneal ulceration did not occur following corneal imaging.

### Case 1

**Fig. 1:**
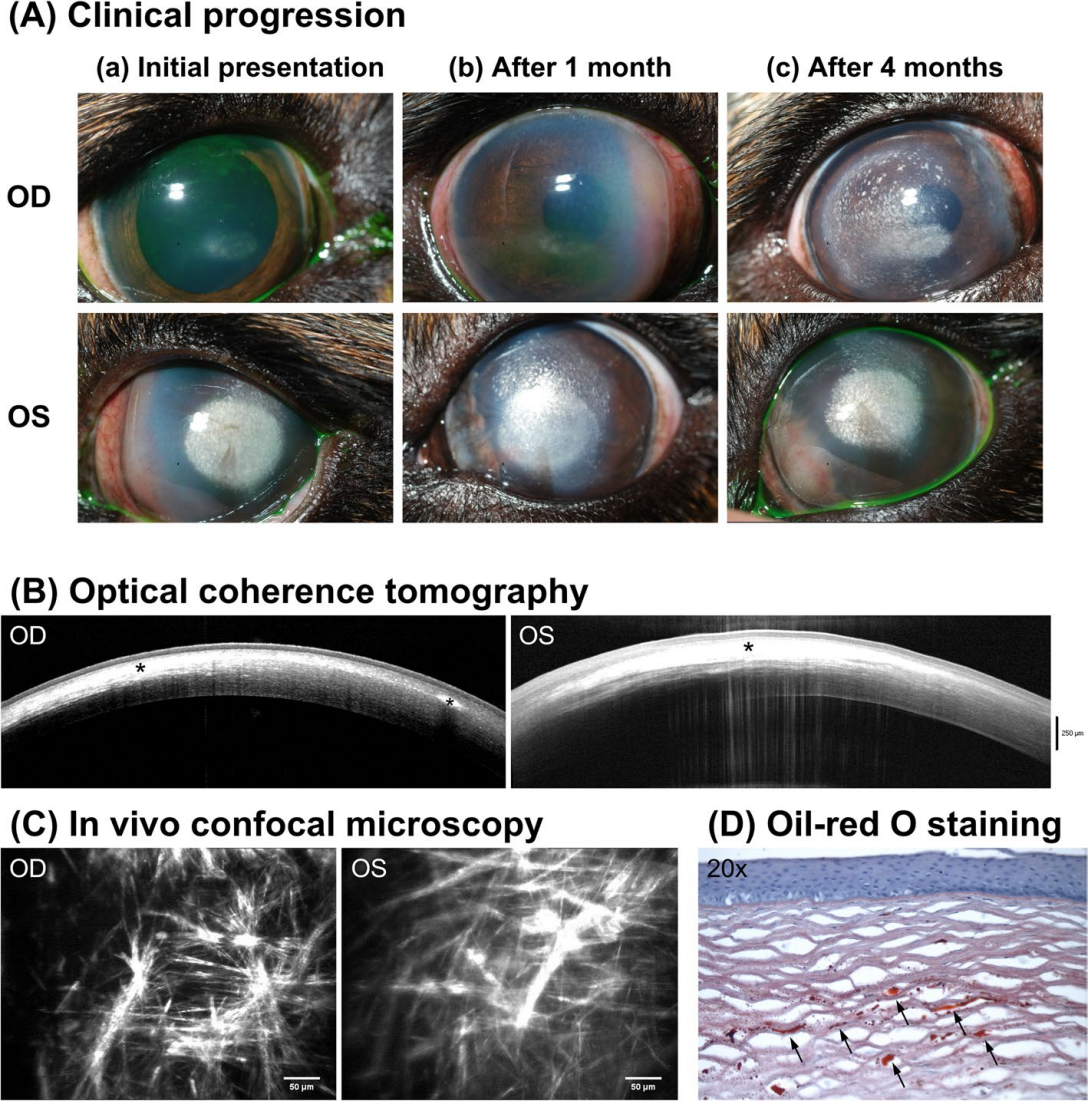
Imaging features of lipid keratopathy in case 1 were consistent with histopathological observations. (A) Clinical progression of case 1, a 4-year-old, male castrated, Cavalier King Charles Spaniel. At initial presentation (a), there was subtle crystalline opacity in the right eye (OD) and dense crystalline opacity with nasal perilimbal infiltration in the left eye (OS). With topical anti-inflammatory treatment, the perilimbal infiltration was resolved OS, but a similar perilimbal infiltration developed in the nasal cornea OD (b). At 4 months after initial presentation and with anti-inflammatory therapy (c), the crystalline opacity was enlarged and more distributed in the cornea OU, but the perilimbal infiltration was resolved OD. (B) Fourier-domain optical coherence tomography (FD-OCT) demonstrated hyperreflective dots or lines along the stromal collagen lamellae from subepithelium to mid stroma in both eyes; these appeared to coalesce into geographic areas of hyperreflectivity (*). Posterior shadowing prevented the view of posterior cornea where the deposits were dense. (C) In vivo confocal microscopy (IVCM) identified highly reflective needle-like structures in both eyes. (D) Oil-red O staining confirmed the presence of lipid deposition which stained red (arrows) in the affected cornea OS

A 4-year-old, male-castrated Cavalier King Charles Spaniel was presented for corneal dystrophy in both eyes (OU). Ophthalmic examinations revealed a horizontally ovoid, crystalline opacity in the axial cornea of the right eye (OD) and a round, dense, white, crystalline opacity in the axial cornea of the left eye (OS) surrounded by anterior stromal punctate deposits in the periphery (Fig. 1 Aa). The medial perilimbal cornea was thickened with tan cellular infiltrates admixed with extensive stromal vascularization OS, which was clinically consistent with episcleritis. The STT-1 were 19 mm/min OD and 9 mm/min OS and IOP were 13 mmHg OU. To improve tear production and episcleritis, neomycin-polymyxin B-dexamethasone ophthalmic suspension and cyclosporine 0.2% ophthalmic ointment were prescribed twice daily OS.

The patient was reexamined one month after initial presentation (Fig. 1 Ab). While the axial corneal opacity OU and the perilimbal inflammation OS were static, a similar perilimbal keratitis developed in the nasal cornea OD despite the medical treatment. The frequency of neomycin-polymyxin B-dexamethasone ophthalmic suspension was increased to 4–6 times daily OU. Following 3 months of medical treatment, perilimbal keratitis was markedly reduced OU, but the axial crystalline opacities had progressed, and a superficial keratectomy was recommended OS (Fig. 1 Ac).

Prior to surgery, FD-OCT and IVCM were performed. With FD-OCT, dense hyperreflective dots or lines were visible along the stromal collagen lamellae and spanned from subepithelium to mid-stroma OU (Fig. 1B). With IVCM, hyperreflective needle-like lines were visible in the affected corneas OU (Fig. 1C). Serum biochemistry including triglycerides, cholesterol, and total T4 concentration were tested; all were within normal limits. Superficial keratectomy was performed at 60% stromal depth as determined by the pre-operative images obtained with FD-OCT. The keratectomy specimen was assessed histologically, and oil-red O staining demonstrated the presence of lipid deposition between stromal lamellae, thus confirming the diagnosis of lipid keratopathy (Fig. 1D).

### Case 2

**Fig. 2:**
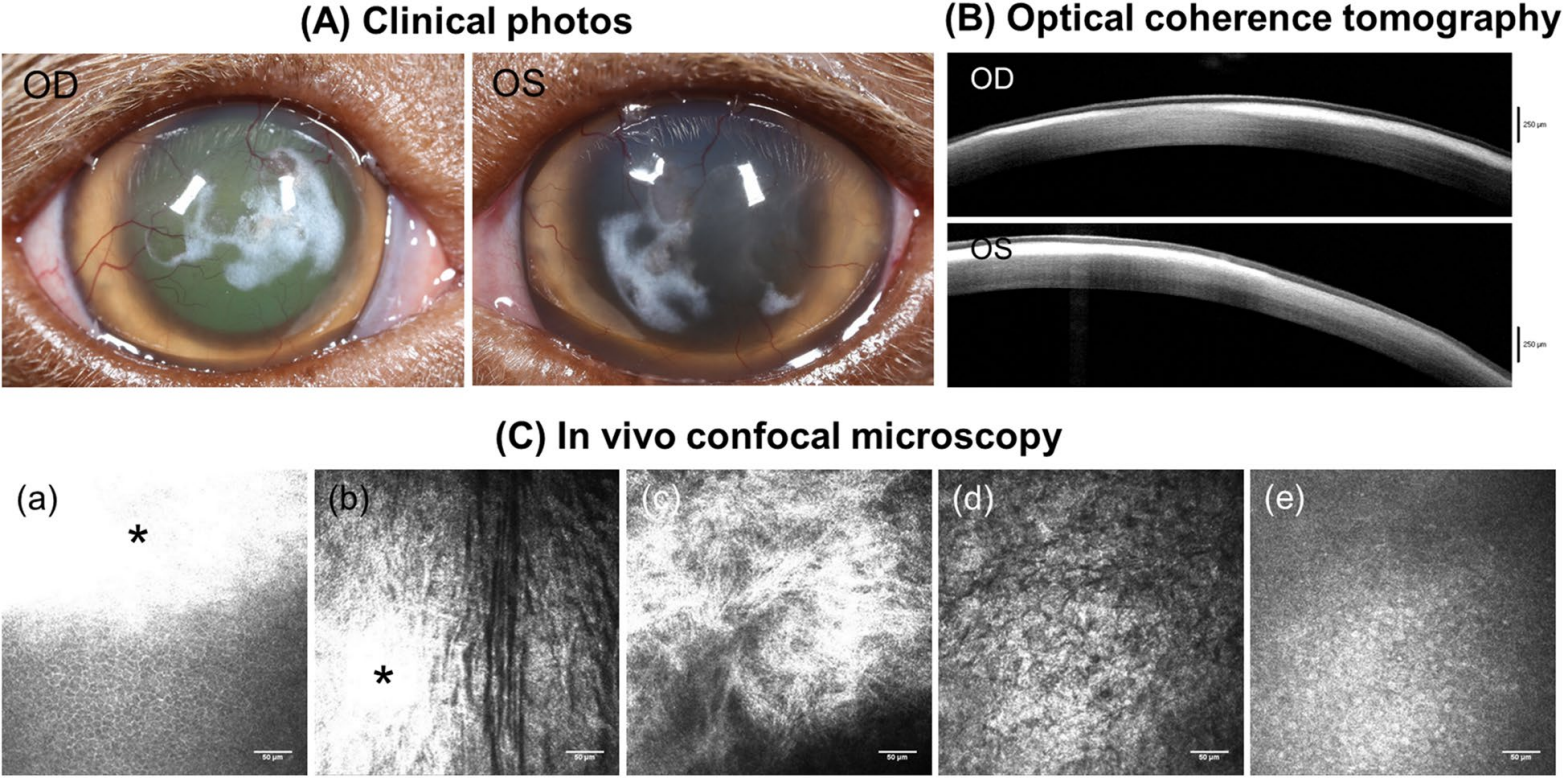
Clinical features of Case 2 affected with mucopolysaccharidosis were in concert with characteristic IVCM findings. Case 2 was a Boston Terrier (female spayed) at 22 months of age. (A) Diffuse stromal cloudiness and chalky white stromal opacity associated with superficial vascularization was identified in both corneas. (B) Fourier-domain optical coherence tomography (FD-OCT) showed dense hyperreflective regions in the anterior corneal stroma in both eyes. Posterior shadowing prevented the view of posterior cornea where the deposits were dense. (C) In vivo confocal microscopy (IVCM) revealed that dense hyperreflective deposits (*) were located in the anterior corneal stroma starting immediately beneath the basal epithelium (a) and distributed multifocally in the cornea (b). Dense hyperreflective deposits appeared comprising of fine shaft or fiber-like structures (c). In the other regions where dense hyperreflective deposits were absent, keratocytes were not observed and corneal stroma appeared diffusely hyperreflective (d), which prohibited visualization of corneal endothelial cells (e)

A 22-month-old, female-spayed, Boston terrier was initially presented to the Neurology service for progressive tetraparesis. According to the owner, this dog could never jump or go down the stairs since the age of 2 months and the owner noticed that the gait and posture had worsened significantly within the past 4 months of presentation. On neurological examination, the dog was mildly obtunded and demonstrated neck pain and postural reaction deficits in all 4 limbs; cranial nerve reflexes were intact and appropriate. On physical examinations, corneal opacity OU was identified as well as audible systolic heart murmur, necessitating consultation with the Cardiology and Ophthalmology service. Ophthalmic examinations revealed diffuse stromal cloudiness in addition to multifocal chalky white opacities in the anterior stroma with superficial vascularization OU (Fig. 2A). Cardiac ultrasound demonstrated moderate aortic valve insufficiency and heterogeneous left ventricular myocardium; blood tests including serum troponin concentration were all unremarkable.

**Fig. 3:**
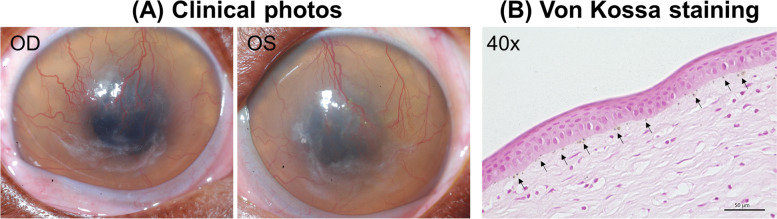
A 15-month-old Boston terrier affected with the same genetic mutation of mucopolysaccharidosis as case 2. **A** Clinical photographs of both eyes taken at 15 months of age. Diffuse stromal cloudiness and subtle white opacity and superficial vascularization were identified in both of his corneas. **B** Von Kossa staining confirmed the presence of calcium deposition in the subepithelium (arrows)

Magnetic resonance imaging revealed moderate ventriculomegaly and multifocal cerebral white matter, thalamic, brainstem and spinal cord T2 weighted and FLAIR hyperintensities without contrast enhancement. These findings including the ophthalmic lesions were consistent with another 15-month-old Boston terrier patient (Fig. 3A) that was presented 2 years prior and diagnosed with MPS type 1 [3]. Case 2 had Sanger sequencing performed which confirmed the same mutation in the alpha-L-iduronidase (IDUA) gene as the previous 15-month-old Boston Terrier [3].

FD-OCT demonstrated a densely hyperreflective band in the anterior stroma associated with occasional irregularly thickened epithelium (Fig. 2B) and IVCM revealed dense, hyperreflective deposits multifocally distributed in the anterior corneal stroma. Dense hyperreflective deposits comprised of fine shaft or fiber-like structures were visible in some regions (Fig. 2 Ca-c) while in others corneal stroma appeared diffusely hyperreflective with no keratocytes visible (Fig. 2 Cd). The constellation of aforementioned hyperreflective stromal opacities limited clear visualization of the corneal endothelium (Fig. 2 Ce).

Although case 2 was euthanized by the primary veterinarian without necropsy, the previous Boston terrier with the same genetic mutation underwent necropsy and the details were described by Mansour et al. [3]. Briefly, affected corneal stroma was infiltrated with CD18-positive macrophages containing abundant vacuoles which were PAS- and Alcian blue-positive indicating glycosaminoglycan (GAG) accumulation [3]. Furthermore, von Kossa stain was implemented to better characterize corneal opacities. Although the white corneal opacities in this prior Boston Terrier (Fig. 3A) were more subtle compared to case 2 (Fig. 2A), von Kossa stain confirmed calcium deposition in the immediate subepithelium (Fig. 3B). In aggregate, the data from these two dogs and a comprehensive review of the physician ophthalmology literature [4–7] suggest that the diffuse stromal hyperreflectivity with the absence of normal keratocytes on IVCM could be a result of generalized GAG accumulation in the stroma and the multifocal, dense hyperreflective deposits observed by FD-OCT and IVCM were most likely calcium deposition from secondary corneal degeneration.

### Case 3

**Fig. 4:**
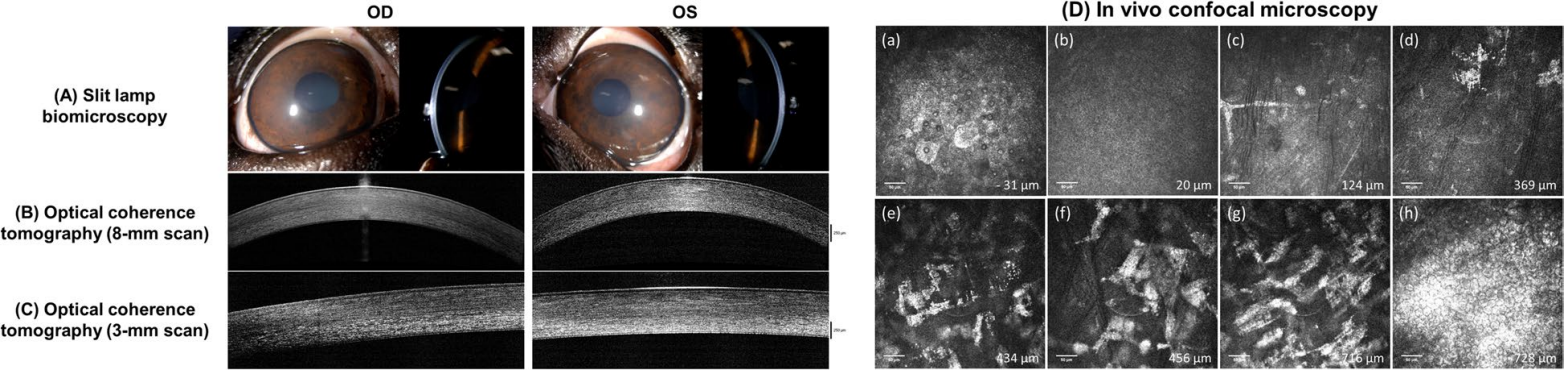
A presumed pre-Descemet corneal dystrophy in case 3 has a unique appearance with corneal imaging. (A) Slit lamp biomicroscopy of case 3, an 11-month-old, male castrated French Bulldog, demonstrated subtle opacity with diffuse illumination and increase reflectivity in the posterior stroma with a narrow-slit beam. (B) and (C) Fourier-domain optical coherence tomography (FD-OCT) using 8-mm and 3-mm scan lengths demonstrated increased reflectivity anterior to Descemet’s membrane with hyperreflective particles scattered in the mid and posterior stroma. (D) In vivo confocal microscopy images (Dimensions mentioned in each image refer to the depth in the cornea at which the image has been acquired). (a) Normal superficial epithelium. (b) Normal basal epithelium. (c) Normal anterior stroma with stromal nerve and keratocytes. (d) Anterior stroma with abnormal keratocytes containing intracellular hyperreflective particles. (e) Mid-stroma with abnormal keratocytes showing extracellular and intracellular hyperreflective particles. (f) Mid-stroma with enlarged abnormal keratocytes containing intracellular hyperreflective particles. (g) Posterior stroma with abnormal keratocytes showing extracellular and intracellular hyperreflective particles. (h) Normal endothelium

An 11-year-old, male-castrated French Bulldog was referred for diffuse posterior corneal opacities OU. The dog was non-ambulatory due to hindlimb paresis from intervertebral disc disease but was otherwise healthy. On ophthalmic examination, the entire posterior stroma was diffusely hyperreflective OU with a narrow slit-beam (Fig. 4A). STT-1 values were 22 mm/min OD and 19 mm/min OS and IOP were 22 mmHg OD and 19 mmHg OS. With FD-OCT, a well-demarcated zone of increased reflectivity anterior to Descemet’s membrane was identified OU; Hyperreflective deposits were diffusely scattered in the middle to posterior stroma and was most dense in the axial cornea (Fig. 4B-C). The corneal epithelium, anterior stroma, and endothelium were normal OU with IVCM (Fig. 4D a-c and h) but punctate, hyperreflective deposits were observed mostly in the keratocytes and rarely in the extracellular matrix. These deposits increased in density from the middle to posterior corneal stroma with more keratocytes affected in the posterior versus middle stroma; some affected keratocytes were larger in size than unaffected keratocytes (Fig. 4D d-g).

The dog had been routinely assessed by a regular veterinarian and complete blood counts and serum biochemistry with cholesterol concentration performed 5 months earlier, all of which were within normal limits. Furthermore, this dog did not show any dermatological signs including alopecia, seborrhea, scaling, and hyperpigmentation commonly manifested by endocrine disorders. Given the symmetry of the corneal opacities in both eyes and non-inflammatory avascular nature, this dog was diagnosed with presumed pre-Descemet corneal dystrophy (PDCD) OU.

## Discussion and conclusions

Both FD-OCT and IVCM provided high-resolution images of corneal layers. It is known that light obstruction by corneal opacities and light scattering by corneal edema can affect the quality of OCT imaging particularly for the posterior cornea [8–10]. Indeed, OCT imaging of cases 1 and 2 showed posterior shadowing where the lipid or calcium deposits, respectively, were dense enough to hinder the path of infrared light emitted by the OCT (Figs. 1B and 2B). It is known that this optical limitation could be overcome by high-frequency ultrasound, which does not depend on corneal transparency [8]. Despite these limitations, IVCM facilitated microstructural evaluation at the cellular level comparable to that of histopathology. Interestingly, the confocal appearance of lipid keratopathy closely resembled histopathological descriptions of corneal lipid deposits [11], which are typically seen as needle-shaped, acicular clefts containing free cholesterol between stromal lamellae on histology [12]. Indeed, en face view of the crystalline opacity on IVCM demonstrated needle-like hyperreflective lines which appeared to be situated along the stromal collagen lamellae as observed with FD-OCT in case 1. By contrast, presumptive calcium deposition in case 2 appeared much denser and plaque-like than lipid deposition. In humans, corneal calcification begins as basophilic granules at Bowman’s layer, which merges and consolidates into solid plaques over time [13].

MPS type 1 is caused by a deficiency of IDUA, a lysosomal enzyme that hydrolyzes intra- and extracellular GAGs [14, 15]. In the absence of IDUA, the affected corneal stroma accumulates GAGs within the cytoplasm and in the extracellular matrix, leading to disorganization of stromal collagen fibrils and fibrosis [15]. Corneal manifestation in dogs affected with MPS type 1 begins with a bilateral ground glass appearance at ~ 6 months of age [14], and severity of GAG accumulation and resultant diffuse stromal cloudiness increases over time [16]. Most of the current literature describing disease progression and investigating novel enzyme and gene therapy for the treatment of corneal MPS type 1 utilized a research dog colony of Plott Hound-Beagle crosses, which are homozygous for a null mutation in IDUA, specifically by the retention of intron 1 in the IDUA gene caused by a mutation in the donor splice site [3, 15, 16]. Despite slight differences in the causal genetic mutations, all MPS type 1-affected corneas undergo secondary degeneration associated with corneal neovascularization and multifocal subepithelial deposits suggestive of calcium in advanced chronic stages [15, 16]. This is consistent with our findings in case 2 that was 22 months old and presented with a more obvious chalky white opacity (Fig. 2A) versus the subtle white opacity observed in the prior Boston terrier that was only 15 months old at the time of presentation (Fig. 3A). Given that GAG-induced inflammation was demonstrated to be involved with the pathogenesis of MPS-related cardiovascular diseases in dogs [17], we assume a similar inflammatory response could occur secondarily to GAG accumulation in the cornea, causing corneal calcium deposition in MPS-affected dogs.

Notably, generalized hyperreflectivity in the corneal stroma inhibited detailed visualization of corneal stromal and endothelial cells by IVCM in case 2. According to the histopathological examinations in MPS type 1-affected canine corneas, there was marked infiltration of GAG-laden macrophages in the entire stroma [3, 16]. While IVCM can detect individual macrophages in rat corneas [18] and few stromal cells with intracytoplasmic inclusions suggestive of GAGs in MPS-affected human corneas [4], we were unable to detect any vacuole-containing, macrophage-like cells or keratocytes in case 2. The diagnostic sensitivity of corneal imaging appears to be poor in identifying pathognomonic changes in the corneal stroma specific to MPS but the lack of keratocytes combined with hyperreflective stroma can increase the index of suspicion for this condition.

It is interesting to note that diffuse stromal hyperreflectivity with depletion of keratocytes in the anterior and posterior stroma with IVCM were consistent with human MPS cases [4–7]. It was suggested that increased reflectivity of corneal stroma observed in MPS patients could be related to scarring/fibrosis or irregular lamellar arrangement of stromal collagen due to GAG accumulation [4–6]. As there is a lack of clear consensus with regards to distinguishing fibrosis, scarring, or extracellular deposition by IVCM [4], definitive diagnosis of MPS should be based on detection of GAG metabolites in urine, enzyme activity assays and genetic testing particularly when histopathology and immunolabeling to confirm GAG accumulation are not feasible.

Nevertheless, corneal imaging could still play a role in discovering unknown or under-recognized pathology in select cases. Interestingly, the imaging findings identified in case 3 have numerous similarities to pre-Descemet corneal dystrophy (PDCD) in humans. The hyperreflective particles scattered in the middle-posterior stroma identified with FD-OCT are consistent with those found in PDCD-affected humans [19, 20]. Hyperreflective keratocyte intracellular/extracellular deposits increasing in density towards the posterior stroma and enlarged keratocytes are characteristic IVCM features of PDCD in humans [19–21]. As PDCD does not affect vision thereby not requiring surgical intervention, the pathology of this disorder is neither well-established nor clearly confirmed hereditary despite its classification as a dystrophy in humans [22]. An ultrastructural study of a human PDCD case revealed enlarged posterior stromal keratocytes filled with numerous membrane-bound intracellular vacuoles containing lipofuscin-like lipoprotein, suggesting that an age-related degenerative disorder is also possible [23].

In conclusion, we demonstrated that corneal imaging could provide instantaneous microstructural analysis comparable to histopathology and differential imaging features could aid in vivo in-depth characterization for under-recognized conditions. Inherent limitations exist for certain disease conditions where the ocular imaging appearance is non-specific. For example, GAG accumulation in the keratocytes of the stroma cannot be observed in MPS. Thus, corneal imaging cannot completely replace current gold standards for diagnosis such as genetic testing and histopathology.

## Data Availability

All data generated or analyzed during this study are included in this published article.

## Abbreviations

FD-OCT: Fourier-domain optical coherence tomography
GAG: Glycosaminoglycan
IDUA: alpha-L-iduronidase
IOP: Intraocular pressure
IVCM: in vivo confocal microscopy
MPS: Mucopolysaccharidosis
OD: Oculus dexter, right eye
OS: Oculus sinister, left eye
OU: Oculus uterque, both eyes
PDCD: Pre-Descemet corneal dystrophy
STT: Schirmer’s tear test

## References

[1] Ledbetter EC, Gilger BC, Gelatt KN, Gilger BC, Kern TJ (2013). Diseases and surgery of the canine cornea and sclera. Veterinary ophthalmology. 5th edn.

[2] Alzubaidi R, Sharif MS, Qahwaji R, Ipson S, Brahma A (2016). In vivo confocal microscopic corneal images in health and disease with an emphasis on extracting features and visual signatures for corneal diseases: a review study. Br J Ophthalmol.

[3] Mansour TA, Woolard KD, Vernau KL, Ancona DM, Thomasy SM, Sebbag L (2020). Whole genome sequencing for mutation discovery in a single case of lysosomal storage disease (MPS type 1) in the dog. Sci Rep.

[4] Grupcheva CN, Craig JP, McGhee CNJ (2003). In vivo microstructural analysis of the cornea in Scheie’s syndrome. Cornea..

[5] Aragona P, Wylegala E, Wroblewska-Czajka E, Smedowski A, Nowinska A, Roszkowska AM (2014). Clinical, confocal, and morphological investigations on the cornea in human mucopolysaccharidosis IH-S. Cornea..

[6] Karaküçük Y, Bozkurt B, Şahin M, Okudan S (2020). In vivo confocal microscopy and anterior segment optical coherence tomography findings in two cases with mucopolysaccharidoses. Turkish J Ophthalmol.

[7] Matoba A, Oie Y, Tanibuchi H, Winegarner A, Nishida K (2020). Anterior segment optical coherence tomography and in vivo confocal microscopy in cases of mucopolysaccharidosis. Am J Ophthalmol Case Rep.

[8] Famose F (2014). Assessment of the use of spectral domain optical coherence tomography (SD-OCT) for evaluation of the healthy and pathological cornea in dogs and cats. Vet Ophthalmol.

[9] Thomasy SM, Cortes DE, Hoehn AL, Calderon AC, Li JY, Murphy CJ (2016). In vivo imaging of corneal endothelial dystrophy in Boston terriers: a spontaneous, canine model for fuchs’endothelial corneal dystrophy. Investig Ophthalmol Vis Sci.

[10] Shull OR, Reilly C, m., Davis LB, Murphy CJ, Thomasy SM. (2018). Phenotypic characterization of corneal endothelial dystrophy in German shorthaired and wirehaired pointers using in vivo advanced corneal imaging and histopathology. Cornea..

[11] Ghanem RC, Ghanem VC, Victor G, Alves MR (2012). Bilateral progressive idiopathic annular lipid Keratopathy. Case Rep Ophthalmol Med.

[12] Dubielzig RR, Ketring K, McLellan GJ, Albert DM (2010). Diseases of the cornea and sclera. Veterinary Ocular Pathology: A Comparative Review.

[13] Ximenes KF, Vasconcelos KFX, Monte FQ (2017). Calcium deposits in the cornea: Histopathological study. Rev Bras Oftalmol.

[14] Shull RM, Munger RJ, Spellacy E, Hall CW, Constantopoulos G, Neufeld EF (1982). Canine α-L-iduronidase deficiency. A model of mucopolysaccharidosis I. Am J Pathol.

[15] Miyadera K, Conatser L, Llanga TA, Carlin K, O’Donnell P, Bagel J (2020). Intrastromal gene therapy prevents and reverses advanced corneal clouding in a canine model of Mucopolysaccharidosis I. Mol Ther.

[16] Newkirk KM, Atkins RM, Dickson PI, Rohrbach BW, McEntee MF (2011). Ocular lesions in canine mucopolysaccharidosis I and response to enzyme replacement therapy. Investig Ophthalmol Vis Sci.

[17] Khalid O, Vera MU, Gordts PL, Ellinwood NM, Schwartz PH, Dickson PI (2016). Immune-mediated inflammation may contribute to the pathogenesis of cardiovascular disease in mucopolysaccharidosis type I. PLoS One.

[18] Peebo BB, Fagerholm P, Traneus-Röckert C, Lagali N (2011). Time-lapse in vivo imaging of corneal angiogenesis: the role of inflammatory cells in capillary sprouting. Investig Ophthalmol Vis Sci.

[19] Malhotra C, Jain AK, Dwivedi S, Chakma P, Rohilla V, Sachdeva K (2015). Characteristics of pre-descemet membrane corneal dystrophy by three different imaging modalities-in vivo confocal microscopy, anterior segment optical coherence tomography, and scheimpflug corneal densitometry analysis. Cornea..

[20] Alafaleq M, Georgeon C, Grieve K, Borderie VM. Multimodal imaging of pre-Descemet corneal dystrophy. Eur J Ophthalmol. 2020;30(5):908-916. 10.1177/1120672119862505 31298040

[21] Shi H, Feng QX, Tao LT, Hao Q, Hong LX, Ling LL (2017). In vivo confocal microscopy of pre-Descemet corneal dystrophy associated with X-linked ichthyosis: a case report. BMC Ophthalmol.

[22] Weiss JS, Møller HU, Aldave AJ, Seitz B, Bredrup C, Kivelä T (2015). IC3D classification of corneal dystrophies-edition 2. Cornea..

[23] Curran RE, Kenyon KR, Green WR (1974). Pre-descemet’s membrane corneal dystrophy. Am J Ophthalmol.

